# Predicting 10-Year Risk of Fatal Cardiovascular Disease in Germany: An Update Based on the SCORE-Deutschland Risk Charts

**DOI:** 10.1371/journal.pone.0162188

**Published:** 2016-09-09

**Authors:** Viktoria Rücker, Ulrich Keil, Anthony P Fitzgerald, Uwe Malzahn, Christof Prugger, Georg Ertl, Peter U Heuschmann, Hannelore Neuhauser

**Affiliations:** 1 Institute of Clinical Epidemiology and Biometry, University of Würzburg, Würzburg, Germany; 2 Institute of Epidemiology and Social Medicine, University of Münster, Münster, Germany; 3 Department of Epidemiology and Public Health, Department of Statistics, University College Cork, Cork, Ireland; 4 Clinical Trial Center, University Hospital Würzburg, Würzburg, Germany; 5 Institute of Public Health, Charité ‒ Universitätsmedizin Berlin, Berlin, Germany; 6 Department of Medicine I, University Hospital Würzburg, Würzburg, Germany; 7 Comprehensive Heart Failure Center Würzburg, University Würzburg, Würzburg, Germany; 8 Department of Epidemiology and Health Monitoring, Robert Koch Institute Berlin, Berlin, Germany; 9 DZHK (German Center for Cardiovascular Research), partner site Berlin, Berlin, Germany; Azienda Ospedaliero Universitaria Careggi, ITALY

## Abstract

Estimation of absolute risk of cardiovascular disease (CVD), preferably with population-specific risk charts, has become a cornerstone of CVD primary prevention. Regular recalibration of risk charts may be necessary due to decreasing CVD rates and CVD risk factor levels. The SCORE risk charts for fatal CVD risk assessment were first calibrated for Germany with 1998 risk factor level data and 1999 mortality statistics. We present an update of these risk charts based on the SCORE methodology including estimates of relative risks from SCORE, risk factor levels from the German Health Interview and Examination Survey for Adults 2008–11 (DEGS1) and official mortality statistics from 2012. Competing risks methods were applied and estimates were independently validated. Updated risk charts were calculated based on cholesterol, smoking, systolic blood pressure risk factor levels, sex and 5-year age-groups. The absolute 10-year risk estimates of fatal CVD were lower according to the updated risk charts compared to the first calibration for Germany. In a nationwide sample of 3062 adults aged 40–65 years free of major CVD from DEGS1, the mean 10-year risk of fatal CVD estimated by the updated charts was lower by 29% and the estimated proportion of high risk people (10-year risk > = 5%) by 50% compared to the older risk charts. This recalibration shows a need for regular updates of risk charts according to changes in mortality and risk factor levels in order to sustain the identification of people with a high CVD risk.

## Introduction

Current guidelines on prevention of cardiovascular disease (CVD) such as the European Guidelines on cardiovascular disease prevention in clinical practice [1], recommend to guide decisions to take preventive actions as well as the level of preventive actions (e.g. lifestyle recommendations, drug treatment) by an estimation of absolute risk of CVD based on multiple cardiovascular risk factors [2, 3]. Since CVD prevention is not only an individual concern but also a worldwide public health concern, there is a need for a common, albeit locally specific risk assessment that can take account of regional disparities in CVD risk as well as of time trends in CVD mortality and CVD risk factors.

As a result, the Third Joint Task Force of European and other Societies on Cardiovascular Disease Prevention in Clinical Practice proposed in 2003 new CVD risk charts for Europe and suggested the SCORE (Systematic COronary Risk Evaluation) project to estimate ten-year risk of fatal cardiovascular disease [4, 5]. The SCORE charts were intended to be calibrated locally which is an advantage compared to non-European based risk equations which considerably overestimate absolute risk in European populations [6–8]. In the following years, risk charts for high-risk and low-risk European countries have been issued and in addition national or regional calibrations of the SCORE risk charts were performed in order to reflect specific mortality and risk factor levels [4, 9–12]. These risk charts should be used for risk estimation in primary prevention only since patients with clinically manifest CVD are already considered as high-risk individuals [1, 4]. In addition, risk can be higher than indicated in the risk charts in patients with specific additional comorbidities (e.g. diabetes) as detailed in CVD prevention guidelines. [1]. Accordingly, in 2005, a recalibrated SCORE-Deutschland risk chart was published based on data for risk factor levels from the German National Health Interview and Examination Survey 1998 (GNHIES98) and the official national mortality statistics of 1999 from the Federal Statistical Office (Destatis) [13]. Since then, decreasing trends in major CVD risk factors such as decreasing blood pressure and cholesterol levels [14, 15] and to a lesser extent smoking prevalence [16] have been observed in Germany, as well as a marked decrease in CVD mortality rates [17]. These trends suggested the need for an update of the risk charts for Germany in order to avoid overestimation of risk and false positive categorization of people from the general population into the high risk group.

Therefore, we updated the calibration of the risk charts based on the SCORE methodology for Germany using risk factor profiles from the most recent German National Health Examination survey (DEGS1 study 2008–2011) as well as national CVD mortality rates in 2012. In addition, risk estimates based on the risk charts published in 2005 and the updated ones were compared in a representative sample of the general population in Germany.

## Methods

### Score project

The first SCORE risk charts for Europe were published in 2003 [4]. The SCORE Project pooled data from 12 European prospective cohort studies from the years 1967–1991 including the German MONICA-Augsburg cohort [18]. The SCORE dataset contains information from 205,178 persons (43% women and 57% men) representing 2.7 million person years of follow-up and 7,934 documented cardiovascular deaths. Based on this dataset, risk charts for cardiovascular mortality were developed in 2003 predicting the absolute risk of dying from CVD in the next ten years. The risk was modelled as a function of age, sex, systolic blood pressure (SBP), cholesterol (total (TC) or total cholesterol/HDL-ratio (TC/HDL-C)) and smoking. Two SCORE risk charts were developed, one for European populations at a high CVD risk (SCORE-high) and one for European populations at a low CVD risk (SCORE-low). For a more precise prediction of risk estimates, calibration of the risk charts according to risk factor and mortality levels of respective countries was proposed [4].

### First calibration for Germany

The first calibration for Germany was published in 2005 [13]. The SCORE-Deutschland risk charts were based on three terms: a German specific baseline absolute risk of death from CVD, relative risks based on the estimates from the SCORE Project and estimates of average risk factor levels of SBP, cholesterol and smoking. The calculations of the SCORE-Deutschland risk charts were based on a Cox proportional hazards model with age as a time variable with delayed entry. Both TC and TC/HDL-C based SCORE-Deutschland risk charts were made available. Previous analyses of the SCORE data had shown similar performance of the TC and the TC/HDL-C based charts and risk estimation with any of the two versions had been recommended in the first German SCORE calibration [13]. The calculations of the absolute risk for the SCORE-Deutschland risk charts published in 2005 were calibrated using the official mortality rates of the year 1999 and risk factor data from the GNHIES98 [19, 20]; the relative risks were derived from the SCORE dataset [4].

### Updated calibration for Germany

For the updated calibration of 10-year risk of CVD based on the SCORE methodology, recent national estimates of age- and sex-specific mean levels of the risk factors SBP, smoking and cholesterol (TC or TC/HDL-C) from the DEGS1 2008–2011 study were used. The investigations of the DEGS1 study were carried out in accordance with the Declaration of Helsinki, including written informed consent of all participants. All study methods were approved by the ethics committee of Charité–Universitätsmedizin Berlin and the federal data protection and deral data protection and freedom of information authority. The examination sample of DEGS1 included 7.115 participants aged 18–79 years from a Germany-wide two-stage clustered sample from local population registries with standardized measurements and interviews [21]. TC and high density lipoprotein cholesterol (HDL-C) were measured using an enzymatic assay (Architect ci8200, Abbott, Germany). Non-HDL-C was calculated by subtracting HDL-C from TC. Three blood pressure (BP) measurements were taken at the right arm at 3-minute intervals after a non-strenuous part of the examination and an additional 5-minute rest with an automated oscillometric device (Datascope Accutorr Plus, Mahwah, NJ). Participants sat on a height-adjustable chair, back supported, arm lying on a table at heart level, feet on the floor and legs uncrossed. Three different cuff sizes were used. The second and third measurements were averaged [14]. Smoking was defined as current self-reported smoking. In order to account for the unequal sampling probabilities related to the sampling design and nonresponse, statistical analyses were weighted using a weighting factor [21] and the complex survey sampling was taken into account using complex samples procedures in SPSS version 20.0 (SPSS Inc., Chicago, Illinois, USA).

The updated calibration also used recent mortality data from Germany. Sex-stratified and 5-year age aggregated number of deaths from CVD and non-CVD (CVD: ICD-10 I10-I15, I20-I25, I44-I51 and I61-I73) and the number of inhabitants from the year 2012 were collected from official mortality statistics. We used competing risk model that utilizes the cumulative incidence function to calculate the average absolute risk of dying from CVD. The competing risks model allows for the fact that there are competing causes of death that influence the actual likelihood of dying from CVD. Non-CVD death was considered as being the competing event and death from CVD the event of interest. It was assumed that the relative risks of the respective risk factors didn’t change over the years. Therefore, relative risks for CVD mortality were derived from an analysis of the entire SCORE database [4]. The statistical method for the updated calibration is based on the methodology introduced previously [13] but was slightly modified as follows: The calibration from 2005 interpolated the 5-year mortality rates to get the average absolute risk of dying in the next ten years. In the new approach the 5-year mortality rates were held constant. No interpolation was used, because a linear interpolation disagrees with the Markov assumption. Therefore, the estimates of age- and sex-specific mean levels of the risk factors were evaluated with regression models using age in constant 5-year age groups and not as a continuous variable. Thus, separate risk estimations for the 40–45 and for the 45–49 year olds were provided in contrast to the 2005 calibration. As a sensitivity analysis, an independent recalculation of the risk charts using the original approach was performed by one of the authors who was blinded to the current recalibration analysis (APF). Statistical analyses were performed in R 3.1.1. A detailed description of the recalibration methods is provided in the Supporting information (S1 File).

### Changes in 10-year risk of fatal CVD estimation

The change in estimated risk between the first SCORE-Deutschland risk charts published in 2005 and the updated risk charts presented in this paper has been evaluated by applying the two risk charts to a nationwide sample of community-dwelling adults aged 40–65 years from the DEGS1 study in 2008–2011 [21]. Out of 3,470 participants of the DEGS1 study aged 40 to 65 years, 131 (3.8%) were excluded due to missing data on major CVD (self-reported previous diagnoses of coronary heart disease (CHD), stroke and heart failure) or missing data on variables included in SCORE. Of the remaining participants, 8.1% (men 10.9%, women 5.1%) were excluded due to a history of major CVD, leaving a sample of 3,061 participants (unweighted sample size) for estimation of cardiovascular risk (49.5% women, 50.5% men, mean age ± SD 50.9 ± 7.0 years). 10-year risks of fatal CVD were calculated using both the original and the updated calibration methods. For higher precision, risk was directly calculated from the respective formulas not from risk chart readings. High risk was defined according to current guidelines as estimated 10-year risk of fatal CVD of 5% or more. Statistical analyses of DEGS1 data were weighted as described above.

## Results

**Fig 1:**
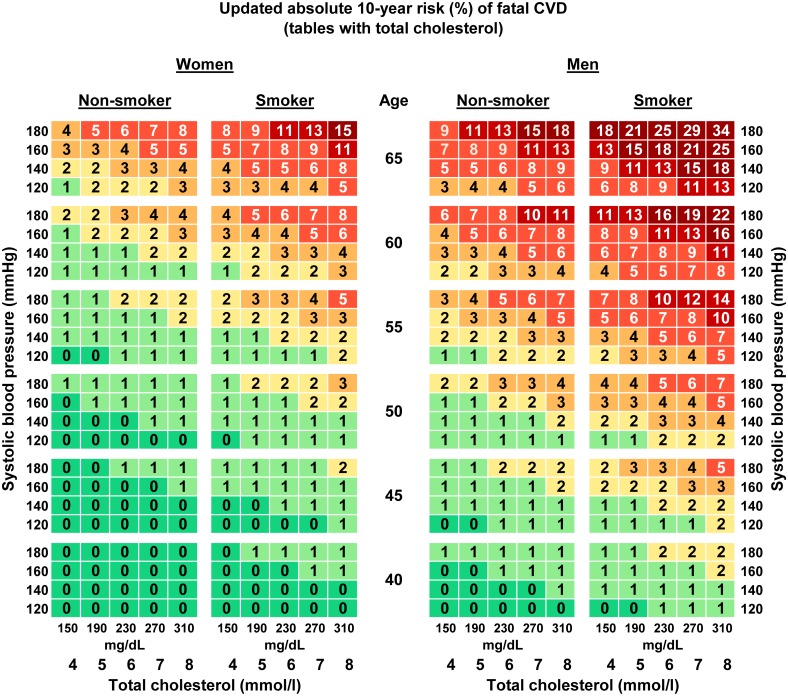
Updated (2015) risk charts for estimation of absolute 10-year risk (%) of fatal CVD in Germany based on the SCORE-Deutschland risk charts (tables with total cholesterol).

**Fig 2:**
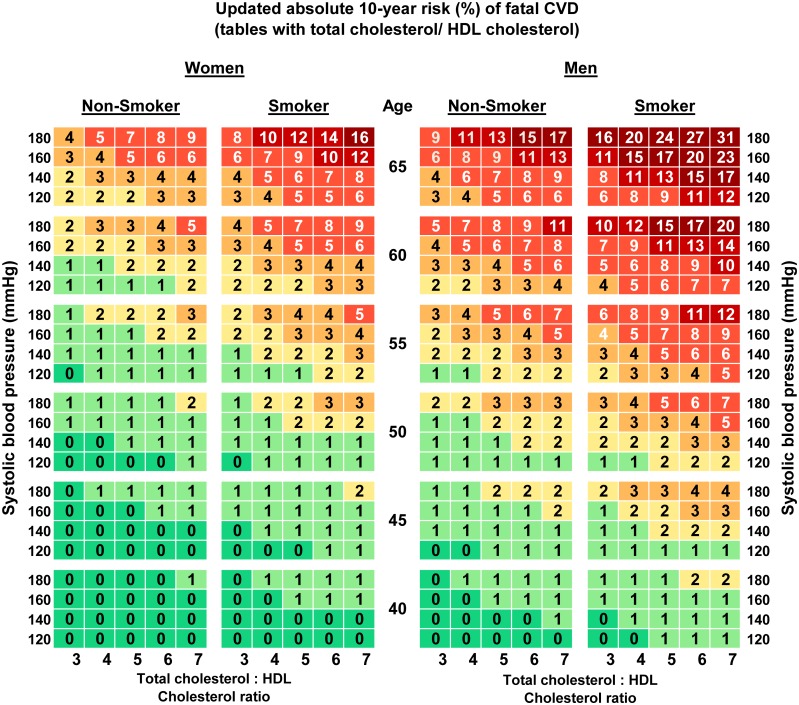
Updated (2015) risk charts for estimation of absolute 10-year risk (%) of fatal CVD in Germany based on the SCORE-Deutschland risk charts (tables with total cholesterol/HDL cholesterol ratio).

The updated risk charts for estimation of the absolute 10-year- risk of fatal CVD in Germany are presented in Figs 1 and 2. Two versions are shown, one using TC (Fig 1) and one using TC/HDL-C (Fig 2). Both versions are stratified by sex and include age, smoking and SBP as additional risk factors. To estimate the absolute 10-year risk of CVD death for an individual, his or her age needs to be rounded to the nearest age shown on the chart and the smoker or non-smoker cell nearest to the person’s SBP and TC or TC/HDL-C ratio will provide the estimated risk.

The updated charts consist of 480 cells. For comparison the cells of the age group 40–49 from 2005 were compared to the cells of age group 40–44 and to the ones of the age group 45–49 from the updated risk chart. Compared to the 2005 predictions in the tables with TC, 34.2% of the absolute cell predictions were higher and 5.6% were lower. For example, the highest absolute risk displayed in a cell of the updated risk charts compared to the one published in 2005 decreased in the TC chart from 37% to 34% for men (absolute risk difference of 3) and from 19% to 15% for women (absolute risk difference of 4) in the highest category for all risk factors. The differences between the old and the updated risk chart for TC in corresponding cells for the age categories 50–65 had a mean of 0.69% (SD +/- 0.72). For the tables with TC/HDL-C, 47.1% of the absolute risks estimated for Germany decreased in comparison with the 2005 risk chart [13] and the highest absolute risks displayed in a cell had a risk difference of 4 percentage points in the charts for men and 5 for women.

Since a slight adaptation in the calibration method was introduced compared to the first calibration of 2005 (use of constant vs. interpolated 5-year mortality rates), a sensitivity analysis was conducted and the updated calibration was performed using both methods. This sensitivity analysis showed an excellent agreement (intraclass correlation coefficient ICC = 0.99) between the method used in the 2005 calibration and the present approach. Agreement was calculated for the age range 50–65 years (320 cells) since for younger ages the cells do not directly correspond (the first calibration has the age category 40–49, the updated calibration has the two age categories 40–44 and 45–49). The differences between the two methods in corresponding cells had a mean of 0.24% (SD +/- 0.27). The risk predictions using the method from 2005 were always slightly higher than the ones using the method from 2015. The method used in 2005 classified 3 additional cells (out of 320) as high risk compared to the method used in 2015.

**Fig 3:**
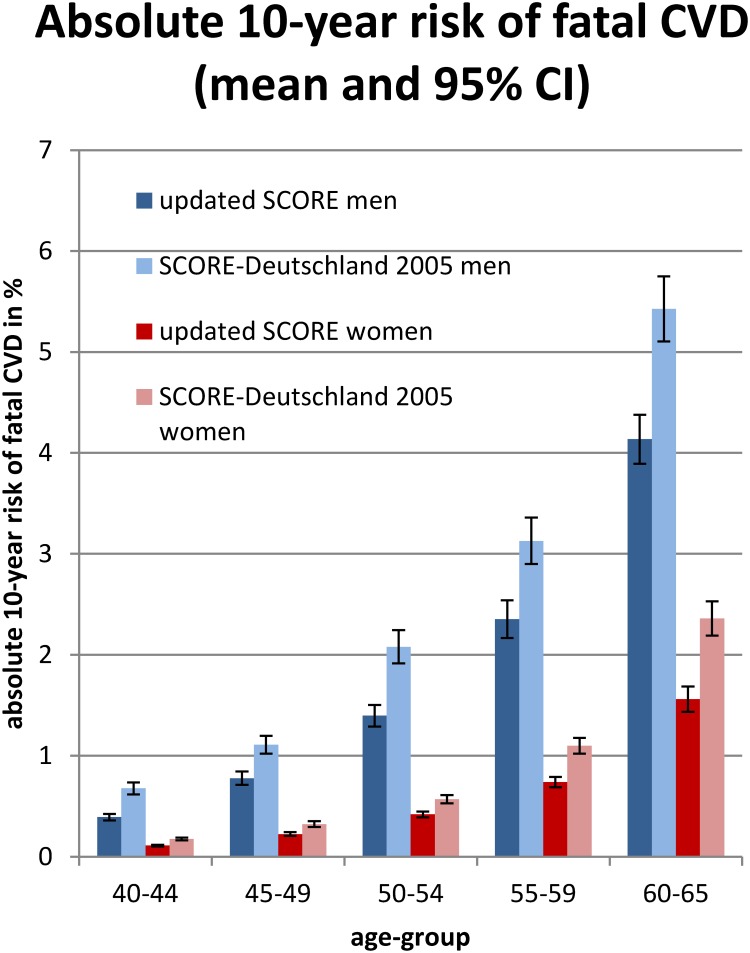
Comparison of absolute 10-year-risk of fatal CVD by age group in adults aged 40 to 65 years in Germany without major CVD according to the first vs. the updated risk charts.

When applied to a representative sample of the population living in Germany aged 40 to 65 years without a previous history of major CVD from the national health survey DEGS1 2008–2011, the updated risk charts using TC classified significantly fewer men and women as high-risk (10-year risk > = 5%) than the German 2005 charts (German risk charts published 2005: 5.2% [95% CI 4.3–6.1]; updated risk charts: 2.6% [95% CI 2.0–3.3]). Average risk was also significantly lower in all age groups among men and women according to the updated charts compared to the charts from 2005 (German risk charts published in 2005: 1.47% [95% CI 1.40–1.54]; updated risk charts 1.04% [95% CI 0.99–1.09]) (Fig 3).

## Discussion

The SCORE risk charts for estimating the absolute 10-year risk of fatal CVD for the German population from 2005 [4, 13] have been updated successfully based on the SCORE methodology [4]. More than a decade after the first calibration of the SCORE charts for Germany [13] these updated charts reflect the decreasing trend in CVD mortality in Germany as well as recent trends towards lower risk factor levels. As expected, the predicted absolute risk generally decreased. The use of the calibrated charts from 2005 for Germany showed a substantially higher population risk in a representative sample of the general population living in Germany aged 40 to 65 years compared to the updated estimates.

Germany has seen a marked decline in cardiovascular mortality in the period between the two risk chart calibrations. The calibration published in 2005 and the updated calibrations are based on mortality data from 1998 and 2012 respectively, a period in which stroke mortality declined by 51%, ischemic heart disease mortality by 45% and overall CVD mortality by 36% (www.gbe-bund.de; mortality rates per 100.000 inhabitants standardized to the population in Germany 2011). In addition, there have been substantial changes in risk factor levels, as documented by the two national health surveys conducted in 1998 and 2008–2011. For example, blood pressure declined considerably (both systolic and diastolic blood pressure by 5 mmHg) [14]. Moreover, cholesterol levels [15] and the proportion of smokers in the German population decreased slightly [16]. However, the fall in CVD mortality might only be partly explained by the changes in risk factors as the chart predicts individual risk at specific risk factors levels and, thus, we wouldn’t expect major changes in the risk chart if the observed decrease in mortality would be entirely explained by risk factor changes.

Numerous risk charts exist for estimating CVD risk. [22] In particular newer risk charts acknowledge the need to issue risk charts for individual countries and recalibrate them regularly [19]. The first recalibration of the risk charts for Germany based on the SCORE project resulted in risk estimates that were between those of SCORE for high-risk and those of SCORE for low-risk countries [13]. The updated risk charts presented in this paper make further adjustments toward lower risk while still lying between SCORE-high and SCORE-low [4]. More than 40% of the 480 cells in the risk charts for Germany are higher and around 1% are lower in comparison to the original SCORE-low risk chart cells [4]. In the updated risk charts the age group 40–49 is divided into two age groups, 40–44 and 45–49 years. This allows for a more precise prediction of the absolute risk. Predicted risk is generally lower according to the updated risk charts compared to the first SCORE-Deutschland risk charts. However, due to rounding of the predicted risk presented in the chart cells rounded predicted risk is numerically the same in more than half of the cells.

Other risk charts are also used in Germany such as the PROCAM score [23], which is based on an occupational cohort from the northwest of Germany, as well as charts like ARRIBA [24] and CARRISMA [25], which are based on adaptations of Framingham risk scores. These charts, however, do not take into account the recent declines in CVD and CVD risk factors in Germany. Results of a German survey on the use of routine risk-scores among physicians showed that more than 50% of the participating physicians believe that risk-scores for CVD are useful for the successful prevention and treatment of CVD [26]. Therefore, it is important that a regular update of the risk-scores based on the current risk factor and mortality distribution of the general population is available.

Our study has certain strengths and limitations. Our recalibration has been confirmed by independent recalculation. The strengths of risk charts based on the SCORE methodology include the large database of European cohorts and the simple and inexpensive use due to restriction to only a few key risk factors. The original SCORE chart combined separate predictions of CHD and stroke mortality.^6^ The method presented here is based directly on national CVD mortality which allows for a more straightforward recalibration. This is of great importance since CVD mortality as well as risk factor levels have been changing considerably in the last decades in many countries [17, 27]. However, two aspects of the SCORE-recalibration may lead to an overestimation of the absolute CVD risk in the general population without preexisting CVD. Ideally, calibration would not require national mortality statistics (which include people with previous CVD who have a higher 10-year-mortality risk than those without a previous CVD), but recent and regional data on mortality of a population free of CVD at baseline. This could be approximated from national mortality statistics with recent and regional CVD incidence data, which, however, are currently lacking. The second aspect is that the competing risk principle is only applied to the country-specific baseline risk but not in the Cox regression to estimate the hazard ratios. This could be solved by using a Fine and Gray Model with competing risks [28] in the 12 original cohorts of the SCORE Project, but is computationally challenging in the presence of left censoring and a model with age as time variable. Finally, it is a general limitation of the SCORE charts that they are limited to fatal CVD prediction and to the age range 40 to 65 years. However, the original SCORE data support robust risk estimation only for fatal CVD and only in this age range. Risk prediction for higher age groups would only be possible by adding cohort data in a higher age range as shown in a recent Norwegian example [29].

### Conclusion

The risk charts presented in this paper update the first SCORE-Deutschland CVD risk charts, based on the SCORE methodology taking into account more than a decade of changes in mortality and risk factor levels in Germany. The absolute ten-year risk of CVD in Germany decreased substantially compared to the risk charts published in 2005. The updated charts allow more exact risk predictions and can help to improve CVD prevention on the population level by a more precise identification of patients with a high risk of CVD.

## Supporting Information

S1 File Recalibration methods. (DOCX)
