# Supplementary material for: Predicting 10-Year Risk of Fatal Cardiovascular Disease in Germany: An Update Based on the SCORE-Deutschland Risk Charts
Source: PLoS One. 2016 Sep 9;11(9):e0162188. doi: 10.1371/journal.pone.0162188 (PMC5017762; doi:10.1371/journal.pone.0162188)
Supplement: S1 File — (DOCX) [file pone.0162188.s001.docx]

**Supporting information (S1 file)**

The following section is based on the supplement of the first SCORE Deutschland calibration [1].^1^The SCORE risk charts predict the absolute risk of dying from CVD in the next ten years. The risk depends on age, sex, systolic blood pressure, cholesterol (total cholesterol or total cholesterol / HDL-ratio) and smoking*.* The risk calculations were stratified for women and men. Different calculations were made based on the total cholesterol and total cholesterol/HDL-ratio. The main principles of statistical analysis will only be explained exemplarily for one sex and for total-cholesterol.

**Description of recalibration**

**Step 1**

First official cardiovascular mortality statistics of Germany for the year 2012 were used to estimate the average probability of CVD death in the next 10-years, M(t), using the competing risks formula. Age is used as the time variable. For the competing risks calculations CVD death was used as the event of interest and non-CVD death was used as the competing event.

$$M\left( t \right)=\frac{\int_{t}^{t+10} \lambda_{cvd}\left( u \right)S\left( u- \right)ⅆu}{S\left( t- \right)}$$

Where λ_cvd_ (t) is the CVD hazard rate for age t and S(t) is the probability of all cause survival till age t. The formula of M(t) can be derived by calculating the probability of dying of CVD before the age of t+10, given that the person is alive at the age of t. All cause survival and CVD hazards were calculated using official cardiovascular mortality statistics of Germany for the year 2012. Cardiovascular deaths are deaths with cause ICD-10: I10-I15, I20-I25, I44-I51 and I61-I73. The mortality statistics were given in 5-year age groups and were stratified for sex. The 5-year mortality incidence rates were being held constant. In contrast to the calibration from 2005, no interpolation was used, because a linear interpolation disagrees with the Markov assumption [2].

**Step 2**

Second, estimates of the risk factors smoking, systolic blood pressure and cholesterol were derived from the up-to-date German Health Interview and Examination Survey for Adults 2008-11 (DEGS1) dataset (Table 1). The estimates of smoking were obtained from logistic regression and the estimates of systolic blood pressure and total cholesterol were derived from linear regression with age as independent variable and stratified for sex.

**Step 3**

The estimate of average 10-year risk of CVD was equated with the risk at average age and sex-specific risk factor levels. The 10-year risk of CVD mortality for someone with specific risk factors levels was calculated based on the proportional hazards model. Calculations utilized the estimated average risk, M(t), the average age and sex-specific risk factor levels, and the log(hazard ratios) from an analysis of the full SCORE database [3, 4].

**Table 1. Estimated average risk factor levels (mean, %) based on DEGS1 data**

| **Sex** | **Age (years)** | **SBP (mmHg)** | **Total cholesterol (mmol/l)** | **TC/HDL ratio** | **Smoking prevalence (%)** |
| --- | --- | --- | --- | --- | --- |
| **Men** |  |  |  |  |  |
|  | 18-19 | 122.84 | 3.97 | 3.18 | 38 |
|  | 20-24 | 125.26 | 4.29 | 3.49 | 48 |
|  | 25-29 | 125.59 | 4.71 | 3.84 | 51 |
|  | 30-34 | 123.69 | 5.13 | 4.26 | 47 |
|  | 35-39 | 125.47 | 5.18 | 4.17 | 37 |
|  | 40-44 | 126.20 | 5.45 | 4.35 | 37 |
|  | 45-49 | 127.92 | 5.39 | 4.17 | 35 |
|  | 50-54 | 130.06 | 5.54 | 4.36 | 38 |
|  | 55-59 | 129.51 | 5.49 | 4.28 | 21 |
|  | 60-64 | 128.29 | 5.35 | 4.13 | 22 |
|  | 65-69 | 129.79 | 5.34 | 4.11 | 14 |
|  |  |  |  |  |  |
| **Women** |  |  |  |  |  |
|  | 18-19 | 112.43 | 4.46 | 3.06 | 42 |
|  | 20-24 | 113.12 | 4.58 | 2.93 | 36 |
|  | 25-29 | 113.87 | 4.76 | 2.97 | 43 |
|  | 30-34 | 111.35 | 4.81 | 3.16 | 33 |
|  | 35-39 | 115.33 | 4.83 | 3.26 | 30 |
|  | 40-44 | 115.59 | 4.97 | 3.14 | 31 |
|  | 45-49 | 120.08 | 5.36 | 3.37 | 34 |
|  | 50-54 | 122.55 | 5.62 | 3.48 | 30 |
|  | 55-59 | 124.94 | 5.87 | 3.61 | 25 |
|  | 60-64 | 126.80 | 5.78 | 3.77 | 19 |
|  | 65-69 | 128.66 | 5.94 | 3.78 | 15 |

**Application**

Suppose we want to estimate the CVD risk for a 57-year-old man, who is a current smoker, with a systolic blood pressure of 160 mmHg, and total cholesterol of 5mmol/l. We have to do the following calculations using the logarithmically transformed hazard ratios, based on an analysis of all cohorts [3].

Based on the DEGS1, the average systolic blood pressure, cholesterol and smoking prevalence among men 55-59 years of age are 129.5 mmHg, 5.49 mmol/l, and 21%, respectively (Table 1). Based on national annual rates for 2012 the competing risks estimate of the 10-year CVD mortality rate for men at the age of 55-59 years is 0.021, thus the corresponding 10-year survival rate is 1-0.021 = 0.979.

1. Calculate k_1_= (5-5.49)* 0.17768 + (160-129.5)* 0.01856 + (1-0.21) * 0.72136 = 1.048
2. Calculate k_2_ = exp(k_1_) = 2.85
3. Calculate k_3_ = 0.979^k2^ = 0.941

So, the 10-year risk of CVD mortality for the above mentioned man is 1-*k_3_* = 1- 0.941 = 0.059 (or 5.9%).

**References**

1. Keil U, Fitzgerald AP, Gohlke H, Wellmann J, Hense H-W. Risk stratification of cardiovascular diseases in primary prevention - Methods. Deutsches Ärzteblatt. 2005;102(25):A1-A2.

2. Gill R, Keilman N. On the estimation of multidimensional demographic models with population registration data. Mathematical Population Studies. 1990;2(2):119-43. doi: 10.1080/08898489009525298.

3. Panagiotakos DB, Fitzgerald AP, Pitsavos C, Pipilis A, Graham I, Stefanadis C. Statistical modelling of 10-year fatal cardiovascular disease risk in Greece: the HellenicSCORE (a calibration of the ESC SCORE project). Hellenic journal of cardiology : HJC = Hellenike kardiologike epitheorese. 2007;48(2):55-63. Epub 2007/05/11. PubMed PMID: 17489342.

4. Conroy RM, Pyorala K, Fitzgerald AP, Sans S, Menotti A, De Backer G, et al. Estimation of ten-year risk of fatal cardiovascular disease in Europe: the SCORE project. European Heart Journal. 2003;24(11):987. PubMed PMID: 146.
